# Nicotinic Receptor Gene *CHRNA4* Interacts with Processing Load in Attention

**DOI:** 10.1371/journal.pone.0014407

**Published:** 2010-12-22

**Authors:** Thomas Espeseth, Markus Handal Sneve, Helge Rootwelt, Bruno Laeng

**Affiliations:** 1 Department of Psychology, Center for the Study of Human Cognition, University of Oslo, Oslo, Norway; 2 Department of Medical Biochemistry, Rikshospitalet, Oslo University Hospital, Oslo, Norway; Université de Montréal, Canada

## Abstract

**Background:**

Pharmacological studies suggest that cholinergic neurotransmission mediates increases in attentional effort in response to high processing load during attention demanding tasks [1].

**Methodology/Principal Findings:**

In the present study we tested whether individual variation in *CHRNA4*, a gene coding for a subcomponent in α4β2 nicotinic receptors in the human brain, interacted with processing load in multiple-object tracking (MOT) and visual search (VS). We hypothesized that the impact of genotype would increase with greater processing load in the MOT task. Similarly, we predicted that genotype would influence performance under high but not low load in the VS task. Two hundred and two healthy persons (age range = 39–77, Mean = 57.5, SD = 9.4) performed the MOT task in which twelve identical circular objects moved about the display in an independent and unpredictable manner. Two to six objects were designated as targets and the remaining objects were distracters. The same observers also performed a visual search for a target letter (i.e. X or Z) presented together with five non-targets while ignoring centrally presented distracters (i.e. X, Z, or L). Targets differed from non-targets by a unique feature in the low load condition, whereas they shared features in the high load condition. *CHRNA4* genotype interacted with processing load in both tasks. Homozygotes for the T allele (N = 62) had better tracking capacity in the MOT task and identified targets faster in the high load trials of the VS task.

**Conclusion:**

The results support the hypothesis that the cholinergic system modulates attentional effort, and that common genetic variation can be used to study the molecular biology of cognition.

## Introduction

Normally, in daily activities we have some control over the choice of stimuli that are allowed to influence our thoughts and actions. That is, a basic cognitive capacity is to focus attention, to shut out distractions, and to persist in search of a solution. The most trivial events require that we constantly invest some cognitive resources and many situations of everyday life are also quite challenging in terms of managing the amount of information that needs to be processed in order to simply keep us alive (e.g., consider navigating among cars, bicycles, pedestrians, and traffic lights and signs when driving through a busy intersection). A mechanism that seems fundamental to these attentive abilities is characterized by the enhancement of one or a few targets, which win nice, clear representation, or depiction, in the brain, and the simultaneous suppression of the remainder “distracting” stimuli, which are consigned to negligible status, if not oblivion. Thus, attention is eminently ‘selective’ and without such a filtering mechanism, our mind would simply be overwhelmed by information or be controlled by irrelevant information. According to cognitive neuroscience models of attention, the selection of the relevant information for access to awareness is achieved by simultaneous attentional enhancement of some objects and suppression of other objects. However, there is more to attention than ‘choosing’ objects of awareness. In ordinary parlance, as the expression *paying attention* suggests, when we focus, we're spending limited cognitive currency that should be wisely invested. According to cognitive psychologist Daniel Kahneman, there are two main classes of traits that define human attention: the *selective* and the *intensive*. The intensive aspect is related to the level of arousal but it actually corresponds best to the phenomenological experience of *effort* or *mental work* or *cognitive load* rather than simply to ‘wakefulness’. Thus, the efficiency of selection depends on the amount of information available as input, implying that attentional capacity is limited [2], [3], [4], [5]. Consequently, performance in attention-demanding tasks depends on both the selective and intensive aspects of attention.

There’s no tidy “attention center” in the brain; instead, cognitive neuroscience studies indicate the existence of an ensemble of alerting, orienting, and executive inter-connected networks in the brain, which all cooperate to the process of concentrating attention on a task [6]. That is, a neurobiology of attention is best described as a distributed functional brain network based on several and parallel neurotransmission systems [1], [7]. Pharmacological studies have provided strong support for the idea that the integrity of the basal forebrain cholinergic system is necessary for normal attentional performance [8]–[16]. Neurons in the Ch4 group of the nucleus basalis of Meynert (nbM), the major cholinergic projection to the cerebral cortex and the amygdala, send axons through the cingulum to the cortex and receives input exclusively from limbic and paralimbic structures. This group of cells can modulate the cholinergic input to the entire cortex based on events of emotional or motivational importance, such as novel or behaviorally relevant sensory events [17].

In addition to the cortical cholinergic pathways originating in the basal forebrain Ch5 of the pedunculopontine nucleus, and Ch6 of the laterodorsal tegmental nucleus, project to the thalamus [18], [19]. Thalamic nuclei play a prominent role in the functional anatomy in several models of attention [20]–[23]. In particular, the pulvinar nucleus of the posterior thalamus has shown activation in functional MRI and positron emission tomography studies on attention [24], [25], and single-cell recordings of pulvinar neurons in monkeys during attention tasks have shown increased firing rate to visual stimuli [26]. It has been suggested that the primary function of the pulvinar in attention is to assign behavioral salience or relevance of visual objects [21], [22], or to function as a priority map by which attentional weights are assigned, which in turn biases a race for selection or representation in visual short term memory [20]. Thalamic nuclei are also implicated in arousal and intensive aspects of attention. Activation of the ascending reticular activation system (ARAS) desynchronizes the cortical electroencephalogram via a cholinergic reticulothalamic pathway [27], [28].

In attention-demanding tasks, cognitive control centers mediate enhancement of task relevant cortical sensory information via prefrontal modulation of cortical cholinergic input [29]. In particular, acetylcholine (ACh) is released in the frontal cortex closely following the time-course of attention-demanding events [30]. According to Kozak et al. [31], increased levels of ACh may be more related to attentional effort than to task performance as such. However, task performance depends on both capacity and attentional effort and effort and capacity are correlated; thus, higher capacity should be associated with low attentional effort. ACh neurotransmission should be associated with task performance since the importance of effort increases when capacity is challenged [1]. In situations where the processing load exceeds the capacity limit, effort should reach an asymptotic level or, with overload, reveal a tendency to reduce [32].

Attentional capacity would seem to widely vary within and between people [2], [3], [5]. Thus, in principle, individual differences in the efficiency of the cholinergic neurotransmission should influence task performance, especially under high task-demands conditions. Single nucleotide polymorphisms (SNPs) in genes coding for ACh receptors typically targeted in pharmacological studies such as the high affinity nicotinic α4β2 receptor could be markers for such individual differences. The α4β2 receptor is the most common nAChR in the human cerebral cortex [33] and is richly expressed in fronto-parietal cortical areas as well as in the thalamus [34]–[37]. In the thalamus the α4β2 nAChR is the only expressed nicotinic receptor [34] with some variation in the expressed distribution of the α and β subunits. In a *Macaca mulatta* localization study [38], α4 mRNA was highly expressed in anterior and dorsal parts of the thalamus as well as the pulvinar, and the signal was particularly strong in the geniculate body, especially the lateral part. The only nucleus without α4 expression was the thalamic reticular formation. The expression distribution of β2 mRNA was similar but weaker, and also included the thalamic reticular nucleus. *CHRNA4*, the gene coding for the α4 subunit in the α4β2 receptor, resides within the richly polymorphic chromosomal area 20q13.2–13.3 [39]. Polymorphisms in exon 5 of the *CHRNA4* gene have been associated with changes in receptor sensitivity in mice [40] and in *xenopus oocyte* models [41]; mutations in exon 5 are known to be associated with rare forms of familial frontal lobe epilepsy [42]. Within exon 5 the common same sense cytosine-to-thymine polymorphism rs1044396 has been shown to influence several traits in humans. Specifically, the T allele has been found to protect against nicotine dependence [43], to be associated with better performance in attention and working memory tasks [44]–[47], and associated with higher cortical responsivity in attention-demanding tasks [48], [49].

In operational terms, attentional effort can be manipulated by systematically changing task demands in perceptual tasks. For example, in multiple object tracking (MOT) tasks participants are asked to pursue with covert attention only (i.e., without moving their gaze from central fixation) a variable number of objects that move independently and unpredictably across the visual display for an extended duration of time (e.g. 10 seconds). This task is thought to allow for measurement of attentional effort over time and therefore is an appropriate test of the ability to sustain attention without interruption. In addition, the task allows the parametric manipulation of processing load by varying the number of objects to be tracked as well as the number of foil objects that need to be ignored. Estimates of maximum tracking capacity are typically of 3–4 objects among an equal or larger number of distracters [50], [51]. However, it is possible to track multiple objects above the classic threshold of subitizing or visual working memory [52].

Importantly, the most commonly used manner in which task demands are increased in attentional experiments is to search for a single target in a visual display among a set of multiple distracters and manipulating perceptual load by either adding the number of visible distracters within the field of vision and/or by varying the shared perceptual features of distracters with the target in visual search tasks [3], [53]–[55]. Therefore, in the present study, we also adapted a visual search task for alphabetic symbols that has been used by Beck & Lavie [56] that consists in searching for a target letter (i.e. X or Z) presented together with another five non-targets and while ignoring centrally another presented distracter (i.e., either an X, Z, or L). Targets differed from non-targets by a unique feature in the low load condition (i.e., O's), whereas they shared features in the high load condition (e.g. K). As cognitive psychology studies with such tasks have repeatedly shown, the first single-feature search differs dramatically from the conjoined-feature search to the point that in the first type of search the target phenomenologically “pops out” regardless of the number of distracters whereas, in the conjoined-feature search, each item in the display is a potential target candidate and consequently search is slow, effortful, and dependent on the amount of distracting elements. In the current study, the main hypothesis is that *CHRNA4* genotype will modulate both visual tracking and visual search performance. Specifically, we hypothesize that the impact of genotype would increase with greater processing load in the MOT task, and that genotype would influence performance under high but not low load in the VS task. Furthermore, we hypothesize that the genetic ‘effect size’ will increase with processing load reaching a peak or to asymptote around the capacity limit. Finally, we will also explore the effects of genotype on distracter processing, given that most of the previous studies have examined more the effects of attentional focusing (on targets) instead of attentional filtering (of distracters).

## Materials and Methods

### Participants

All participants read an information sheet and signed a statement of informed consent approved by the Regional Committee for Medical and Health Research Ethics (South-East Norway) (Project ID: S-03116). Permission to obtain and store blood samples for genotyping, as well as cognitive and MRI data in a biobank, and to establish a registry with relevant information for a time period of 10 years, was given by the Norwegian Department of Health. The research was carried out in compliance with the Helsinki Declaration.

Two hundred and two persons (131 females) in the age range 39–77 (Mean = 57.5, SD = 9.4) participated. All participants were recruited by advertisements in a local newspaper to take part in a larger community based study on the genetics of cognition. All participants were native speakers of Norwegian. Norway has a relatively stable settlement pattern and homogenous population. Participants were not further interviewed about their ancestry, but all participants in the current sample is part of a genome wide association scan for which an extensive quality check has been done with standard procedures implemented in the software package PLINK [57], [58] (Christoforou & Le Hellard, in preparation). All subjects were interviewed and screened for neurological or psychiatric diseases known to affect the central nervous system, and history of substance abuse. Any person with a history of treatment for any of the above was excluded from further participation. The participants were administered the Vocabulary and Matrix reasoning subscales of the Wechsler Abbreviated Scale of Intelligence [59] to estimate general cognitive abilities, and the Beck Depression Inventory (BDI) [60]. Participants included in the study performed within an estimated full scale IQ range of 88 to 148 (Mean = 119, SD = 10.8), and had a total BDI score of 15 or lower. There were no significant or trend level differences between genotype groups on age, sex, IQ, BDI score, or length of education. There were 24 smokers among the participants but there was no association with genotype, χ^2^ = 0.8, *P* = 0.67, with 9, 12, and 3 smokers in the TT, CT, and CC group, respectively.

### Genotyping

Genotyping was performed by real-time PCR with allele-specific fluorescence energy transfer probes and melting curve analyses on the LightCycler™ system (Roche Diagnostics, Mannheim, Germany). DNA was extracted from 300 µL whole blood using MagNA Pure LC DNA Isolation Kit – Large Volume on the MagNA Pure LC (Roche), eluted and diluted to 1 mL, of which 5 µL was applied in each assay. The genotyping was performed at the Section for Genetic Analyses, Department of Medical Biochemistry, Oslo University Hospital, Norway. The analyses were performed in batches of 17–30 samples. The call rate was 100%. No genotyping errors were identified on control repeat analyses or DNA sequencing. Allele frequencies were 0.57 for the T allele and 0.43 for the C allele. There were 62 T allele homozygotes, 105 heterozygotes, and 35 C allele homozygotes, yielding genotype frequencies consistent with the Hardy – Weinberg equilibrium, χ^2^ = 0.071, *P* = 0.4. Details on the typing of the c.1629C>T polymorphism (rs1044396) of the *CHRNA4* gene can be found in [48].

### Tasks and Procedures

#### Multiple object tracking task (MOT)

**Figure 1:**
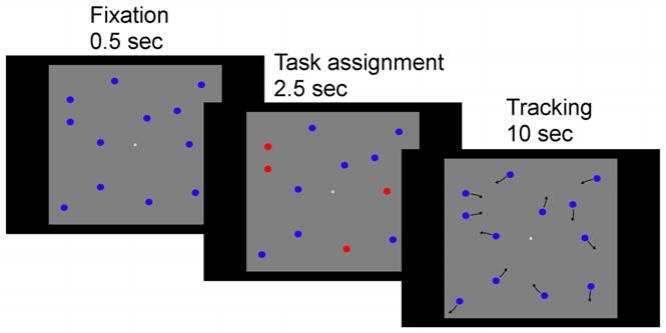
The multiple object tracking task procedure and a selection of trial displays.

Stimuli were presented on a 21″ EIZO CRT monitor using the Psychophysics toolbox extensions (version 3, [61], [62] for MatLab (MathWorks, Natick, MA). Each trial began with the appearance of a centrally presented, white 0.2° diameter fixation point, and twelve blue, 0.7° diameter discs, non-overlapping and randomly spread over the gray 17°×17° display area (see Figure 1). After 0.5 sec a subset of two to six discs turned red for 2.5 sec before returning to blue; the red color designated the target discs to be tracked in the current trial. After a brief interval (0.5 sec) the discs started moving in random directions with a speed of 5.5° per second. To avoid predictable trajectories, each disc made a random change in direction on average once per second. The moving discs bounced off the edges of the display area as well as off each other when they got too close (1°, edge to edge). Additionally, to avoid pulling fixations away from the center the fixation point also “repelled” the discs. After 10 seconds the discs stopped moving and the participant, using the mouse cursor, indicated which objects he/she had been tracking. After clicking on the designated number of target discs, the participant received feedback about the number of correctly tracked targets in the trial. Participants completed five practice trials, one per load condition (number of items to be tracked), before commencing on the experimental trials. Each load condition was presented 20 times in the experiment, which consisted of 100 trials – randomized over conditions. Participants controlled the pace of the experiment by initiating the start of a trial with a mouse click. The experiment typically lasted 35 minutes.

#### Visual search task (VS)

**Figure 2:**
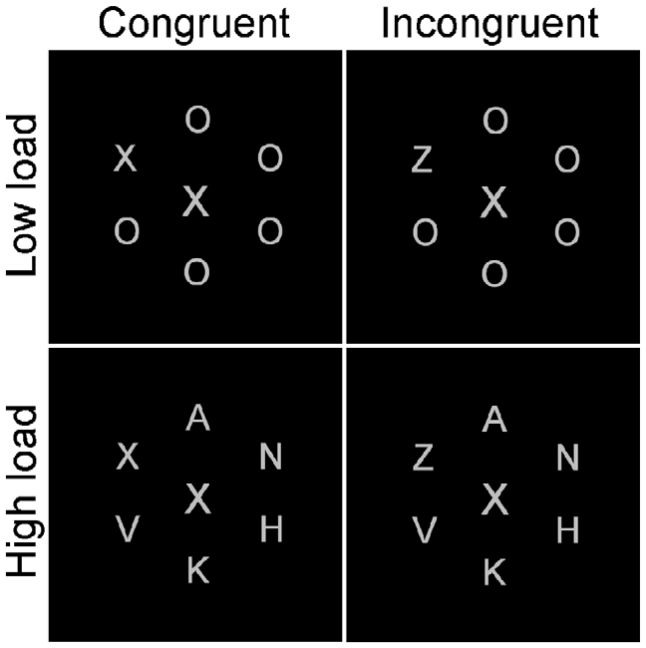
The visual search task displays with high and low load conditions and congruent and incongruent trials (neutral condition display is not shown).

The experiment was run with the E-prime software (Psychology Software Tools, Inc., Pittsburgh, PA; [63], on IBM compatible PCs with 21″ EIZO CRT monitors. Viewing distance was approximately 60 cm. All stimuli were presented in light gray color on a black background (see Figure 2). Target letters (capital *X* or *Z*) appeared randomly but with equal probability in one of six possible positions arranged evenly on a circle with a radius of 2° from the center of the display. Non-targets were presented in the other five positions. In the low perceptual load condition, non-targets were always capital O's. In the high load condition, non-targets were capital *K*, *M*, *H*, *N*, and *V* presented in each of the positions randomly with equal probability. The targets and non-targets subtended 0.36°×0.54° of visual angle. Task-irrelevant distracters subtending 0.43°×0.67° of visual angle were presented centrally on the screen. The identity of the distracter was equally likely to be congruent (e.g. *X* when the target was an *X*), incongruent (e.g. *X* when the target was a *Z*), or neutral (i.e. L when the target was an *X* or a *Z*). Each trial began with a fixation cross presented centrally on the screen for 1000 ms. The circular letter display and distracter were then presented for 100 ms. Participants were asked to ignore the distracter and were told that it was irrelevant to the task and that attending to it might impair their performance. Participants were asked to respond by pressing the leftmost key on a response box with their left index finger if the target was the letter *X*, and by pressing the rightmost key with their right index finger if the target letter was a *Z*. Both speed and accuracy were emphasized. Feedback was given in the form of a short beep for errors or failures to respond within 2 seconds. After a response, or 2 seconds without any registered response, the next trial began. After each experimental block participants were informed on the screen about their average RT and accuracy. There were three low load blocks and three high load blocks. Trial types within each block were counterbalanced and randomized. Each block consisted of 24 trials per congruency condition, yielding a total of 72 congruent, incongruent, and neutral trials per load condition. Half of the participants completed three blocks of the low load condition first and high load second. The other half did the opposite sequence. All participants completed 36 practice trials in which an equal number of low and high load trials were randomly intermixed. The whole experiment was completed in approximately 20 minutes.

## Results

### Multiple object tracking task

**Figure 3:**
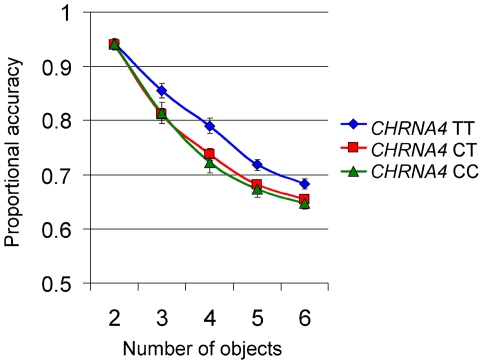
Proportional accuracy on the multiple accuracy task for each of the *CHRNA4* genotype groups over load conditions. Error bars represent standard error of the mean.

**Figure 4:**
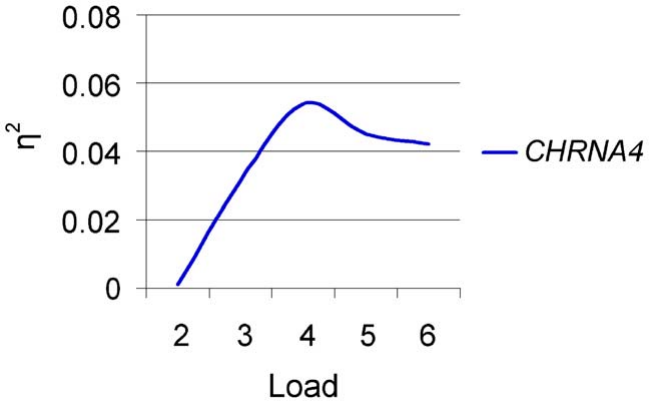
Estimated effect size (partial eta squared) of *CHRNA4* genotype as a function of load condition in the multiple object task.

Mean proportional correct responses were submitted to a repeated-measures ANOVA with Load (2, 3, 4, 5, 6 objects) as within-subjects factor and Genotype (TT, CT, CC) as between-subjects factor. As expected, there was a highly significant main effect of Load, *F*(4, 796) = 836, *P*<0.0005, η^2^_p_ = 0.81. There was also a significant main effect of Genotype, *F*(2, 199) = 4.2, *P* = 0.016, η^2^_p_ = 0.04. Post hoc test with Tukey's HSD revealed that TT carriers had significantly higher accuracy than the two other groups, which did not differ from each other. Crucially, there was a significant Load x Genotype interaction, *F*(8, 796) = 3.6, *P*<0.0005, η^2^_p_ = 0.035. Planned comparisons with one-way ANOVA shows that genotype groups did not differ from each other at the lowest tracking load (i.e. two objects, *P* = 0.95), but was significantly different at all the other tracking loads (*P*'s = 0.043, 0.004, 0.01, and 0.014 for 3–6 respectively, see Figure 3. CT and CC carriers had similar accuracies at all load levels, suggesting a dominant effect of the C allele. Estimated effect size (partial eta squared) of *CHRNA4* genotype on performance was dependent on processing load. Plotting partial effect size over load condition revealed a pattern of linear increase up to a load of four objects, followed by a trend towards a modest reduction (see Figure 4).

### Visual search task (VS)

#### Test-retest reliability

Thirty four participants not part of the sample used in the present study were tested twice on the VS task on average about six months apart. RTs between first and second testing sessions were highly correlated for both the low and high load conditions, *r* = 0.65 and 0.62, and the same was observed for accuracy rates, *r* = 0.56 and 0.54.

#### Accuracy

**Figure 5:**
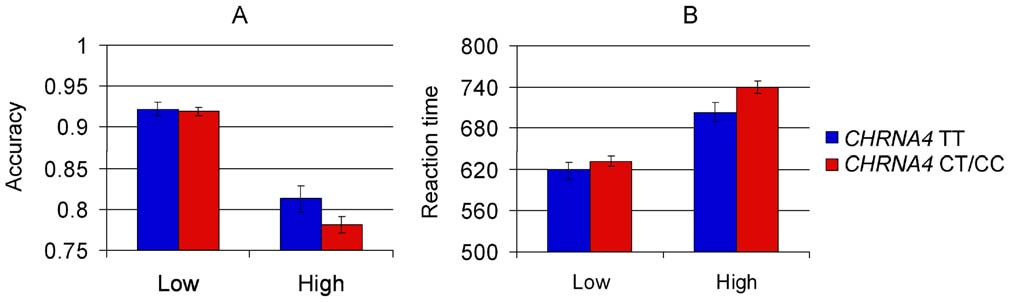
A: Mean accuracy in the visual search task for the *CHRNA4* C- group versus the C+ group as a function of load condition. B: Mean of median reaction time in the visual search task for the *CHRNA4* C- group versus the C+ group as a function of load condition. Error bars represent standard error of the mean.

Mean accuracy was submitted to a repeated-measures ANOVA with Load (low, high), and Congruency (congruent, incongruent, neutral) as within-subjects factors, and Genotype (TT, CT, CC) as between-subjects factor. There was a significant main effect of Load, *F*(1, 199) = 310.5, *P*<0.0005, η^2^_p_ = 0.61, and Congruency, *F*(2, 398) = 46.0, *P*<0.0005, η^2^_p_ = 0.19. Accuracy was higher in low load trials (92%) than in high load trials (79%), and higher in congruent (87%) and neutral (87%) than incongruent (83%) trials. There was no interaction between Load and Congruency, and neither a significant main effect nor an interaction effect involving Genotype. However, the data indicated a dominant effect of the C allele, which is consistent with the results in the MOT task. We therefore ran the analysis again with a dichotomized Genotype factor (C- (TT only) vs. C+ (CT and CC combined)). There was still no main effect of Genotype, but a significant Load x Genotype interaction could now be observed, *F*(1, 400) = 4.0, *P* = 0.046, η^2^_p_ = 0.02. Post hoc tests revealed that TT carriers had better accuracy than C allele carriers under high load (81% vs. 78%), but not low load (92% vs. 92%), consistent with the MOT results (see figure 5A).

#### Reaction time

Median RTs were submitted to a repeated-measures ANOVA with Load (low, high), and Congruency (congruent, incongruent, neutral) as within-subjects factors, and Genotype (TT, CT, CC) as between-subjects factor. There were significant main effects of Load, *F*(1, 199) = 377, *P*<0.0005, η^2^_p_ = 0.66, and Congruency, *F*(2, 398) = 169, *P*<0.0005, η^2^_p_ = 0.46. RTs were slower in high load trials (728 ms) than in low load trials (628 ms), and slower in incongruent (702 ms) than in neutral (670 ms) and congruent trials (663 ms). There was also a significant Load x Congruency interaction, *F*(2, 398) = 34.5, *P*<0.0005, η^2^_p_ = 0.15. Post hoc analyses revealed that the effect of Congruency was significantly larger in low load trials (incongruent – congruent = 53.8 ms) than in high load trials (incongruent – congruent = 24.5 ms), *t*(201) = 7.71, *P*<0.0005.

There was no main effect of Genotype, *F*(1, 199) = 1.6, *P*>0.1, η^2^ = 0.016. However, Genotype interacted significantly with Load, *F*(2, 199) = 3.1, *P* = 0.048, η^2^ = 0.03, but not with Congruency, *F*<1. Post hoc analyses revealed no significant simple effects, but a marginally significant RT difference in the high load condition (*P* = 0.085). Again, the plot indicated a dominant effect of the C allele. Thus, a reanalysis with the dichotomized *CHRNA4* variable (C− vs. C+) revealed a more stable Load x Genotype interaction, *F*(1, 400) = 5.9, *P* = 0.016, η^2^ = 0.03, with a significant simple effect difference in the high load condition, *t*(200) = 2.2, *P* = 0.027, but not the low load condition, *P* = 0.29 (see figure 5B).

## Discussion

Consistent with our primary hypothesis, that *CHRNA4* genotype modulated performance *only* in conditions characterized by intermediate to high processing load in both of our attentional tasks. Specifically, in the MOT task, genotype predicted accuracy only when tracking load was greater than two objects. Moreover, there was a linear increase in the effect size up to four objects, corresponding to the typical capacity limit for the average participant [50], [51], [64], after which the effect size tended to drop slightly. In the VS task *CHRNA4* was associated with higher accuracy and shorter reaction times in the high load condition, but not the low load condition. Although load interacted with the congruency effect as predicted, there was no effect of *CHRNA4* on distracter processing. The combined results of the MOT and VS tasks provide strong support for the notion that nicotinic neurotransmission is involved in the mediation of attentional effort in human cognition.

In both the MOT and VS tasks, there were dominant effects of the C allele. The fact that the pattern of association was stable across tasks possibly indicates common computational underpinnings of these two superficially quite different tasks. The MOT performance parametrically activates core brain attentional nodes and is characterized by on-line continuous perceptual processing with relatively little involvement of visual memory processes. Successful performance in this task also requires sustained attention over time, besides simultaneous division of attention over multiple objects, and continuous active monitoring of their trajectories. The above features make MOT a rather more demanding task (and richer in information) than the most commonly-used sustained vigilance tasks [51]. In addition, the VS task requires identifying briefly presented target letters among non-targets while ignoring distracters. According to several theories on visual search performance, attention is necessary in high load conditions for the binding into a unitary object of perception of the separate perceptual features that are analyzed in parallel by the visual system [54], [55]. As with the MOT task, visual search is also considered to be relatively independent of memory processes [65]. The present results suggest that attentional effort may be a common feature between the two tasks.

Although load strongly modulated the congruency effect in the VS task, *CHRNA4* did not appear to influence the processing of distracters. This may be a surprising result since distracter processing is believed, according to several models of attention, to be a function of both target selection and perceptual load [5], [66]. Indeed, Deco and Thiele [67] speculate that the function of ACh is to enhance the representation of stimuli at the current attentional focus, while simultaneously protecting it against interference from competing stimuli. Moreover, although the low load condition involves feature search in which attentional processes may be minimally employed, it also involves relatively strong requirements for distracter suppression. The distracter was foveally presented and associated with one out of two response alternatives. Thus, although the position of the distracter clearly indicated that its identity was irrelevant, signals resulting from the involuntary processing of this foil stimulus should interfere with task performance. The congruency effect was on average relatively robust in the low load condition (∼54 ms), more than twice as large as the effect in the high load condition (∼25 ms) consistent with previous studies showing that flanker effects are reduced when load increases [3], [56], [68]–[72] but it was nevertheless unrelated to *CHRNA4* genotype. According to Forster and Lavie [73], high load should suppress individual differences in distracter interference effects. The high load congruency effect for the C+ (25 ms) and C− (24 ms) groups are consistent with this view. However, there were no genotype related differences in the low load condition either, with congruency effects of 53 ms and 56 ms for C+ and C− respectively.

Thus, the present results may indicate that selection and distracter suppression are two separate processes that are mediated by separate transmitter systems in the brain. Furthermore, this may suggest that the high affinity α4β2 receptor is not involved in the mediation of distracter suppression, or at least that it may not be involved strongly enough to be detected as a main effect. It could be the case that *CHRNA4* interacts with other markers such as catecholaminergic genes. It is known that α4β2 receptors are found at presynaptic terminals of dopaminergic neurons [74], suggesting that the *CHRNA4* polymorphism alters the affinity of presynaptical nAChRs on dopaminergic neurons. *CHRNA4* was recently found to influence spatial working memory in interaction with the noradrenergic gene *DBH* [45], [46]. Also, in Markett et al. [46] carriers of a *DRD2* TCT+ haplotype had better working memory capacity (Cowan's *K*) than TCT-, but only for *CHRNA4* TT carriers. Moreover, there is by now a substantial literature on the influence of dopaminergic genes such as *COMT, DAT1, DRD2, and MAO1* on executive attention (see ref [75] for an overview). Studies in cognitive neuroscience have indicated that orienting to targets and suppressing distracters are subserved by partly independent functional networks. In particular, fMRI data have shown that the cingulated cortex is activated during the presentation of task-irrelevant distracters or during other types of cognitive conflict [76]–[78]. Several models of attention state that the cingulated cortex is the central node for conflict resolution or conflict monitoring [22], [23], [79]. The anterior cingulated cortex receives input from the ventral tegmental dopamine system and all dopamine receptor subtypes are expressed in layer five of the cingulated cortex [80]. In an early imaging genetics study involving the attention network task flanker elicited BOLD response in the anterior cingulated cortex was modulated by polymorphisms in the dopamine receptor gene *DRD4* and the monoamine oxidase A gene (*MAOA*) [81]. Thus, functional imaging, biochemical, and genetic association evidence indicate that orienting to targets and distracter suppression depends on partly separate functional networks.

Other candidates for interactions are muscarinic receptors [82]. While ACh suppresses the efficacy of excitatory intrinsic connections through muscarinic receptor mechanisms, it enhances thalamocortical synapses preferentially through nicotinic receptors [83]–[85]. Thus, the effect of ACh on task performance in the VS task may involve an interaction of muscarinic and nicotinic receptor activity.

Another possibility might be that ACh related activity does not operate at the time scale of distracter interference (cf. ERP results in [48]). Like the well-known Stroop interference effect, flanker effects may be relatively more related to response conflict rather than perceptual competition. ACh effects may be primarily associated with perceptual processes through phasic bursts of ACh rather than low frequency tonic changes [86].

In visual and auditory oddball tasks, T allele homozygotes have been shown to have higher amplitudes in early ERP components [35]. Also, in an fMRI study with a visual oddball paradigm, T allele homozygotes had stronger BOLD responses in frontoparietal and anterior cingulated cortices [49]. Greenwood et al. [44] used a visual search task in which the location of the target was indicated by use of spatial cues of four different sizes. Larger cues are less precise and will presumably make target identification more challenging. In addition, there were two search conditions; feature search and conjunction search, similar to the VS task used in the present study. *CHRNA4* modulated reaction times in conjunction search only, and the effect seemed to increase with cue size. Does ACh influence effort rather than orienting, scaling, and switching? Depending on the mechanisms behind MOT (e.g. rapid switching between targets [87] or multifocal attention [88]) load may be confounded by spatial area, but VS is not. This may be of relevance to the results of Greenwood et al [44]. Their effect may be an effect of increased effort elicited by larger cues, or a combination of increased effort and spatial scaling, for example by increased effort needed to scale attention properly. In any case, the present MOT results seem to match the Greenwood and colleagues' visual search results exactly, down to the dominant effects of the C allele. According to Greenwood et al [82], and based on Yu & Dayan [89], TT carriers may have greater reactivity to unexpected events, possibly due to increased sensitivity of the receptor. However, in the MOT task, there are no unexpected events, just sustained tracking at temporally separate load conditions, which suggests that mental effort may be a possible mechanism.

While there is converging evidence that exon 5 of *CHRNA4* plays a role in normal attention, neuropsychiatric disorders and nicotine dependence, the rs1044396 C-T substitution in question results in synonymous translation. Using a *xenopus oocyte* model, Hoda et al. [90] measured the electrophysiology of human *CHRNA4* haplotypes, including the rs1044396 SNP, and assessed the density of receptors in high-affinity state associated with the haplotypes. The haplotypes did not differ in terms of electrophysiology, but the haplotype including the C allele differed from the one containing the T allele on the number of receptors in high-affinity state. Thus, although the rs1044396 SNP does not result in a direct amino acid substitution (serine-to-serine in amino acid position 543), it can be associated with altered receptor responsiveness. However, whether the C-T polymorphism is causal, by influencing gene expression via altered binding of regulatory factors, by affecting the splicing pattern, folding or stability of the RNA, or whether the polymorphism merely is linked to another causal polymorphism, has not yet been determined. The rs1044396 SNP is apparently not found in the Chimpanzee genome and is assumed be specific for humans. The C allele is ancestral and is the predominant allele in Sub-Saharan Africa (Yoruba) with a frequency of 97.5%. The C allele is also very common among Han Chinese (82%) but relatively less prevalent among people of Japanese (60%) and European (40%) descent [91]. This raises the possibility that there has been a positive selection of the T allele, possibly as a function of better performance in real life dual task situations such as efficient foraging for “cryptic” food (that is, similar to its surroundings, requiring attention-demanding conjunction search) while staying watchful of potential predators [92].

Nicotine facilitates attentional processes and is also highly addictive. Evans and Drobes [93] suggested that at least a subgroup of cigarette smokers self-administer nicotine to compensate for small attentional deficits. There are by now several reports concluding that *CHRNA4* C allele carriers may have less efficient top-down allocation of attentional resources. The finding that the C allele is associated with increased susceptibility to nicotine dependence [43] may thus suggest that the smoking habits among some nicotine dependent are an indicator of “self-medication”. This may be especially prevalent among patients suffering from schizophrenia [94]. Attentional impairments are believed to be central to the cognitive deficits typically found in patients with this disorder [95], and dysregulation of the basal forebrain cholinergic system may be the source of these effects [29]. Consistent with this, it has been suggested that patients suffering from schizophrenia may smoke to compensate for cognitive deficits [96], [97].

Chronic exposure to nicotine through smoking has significant and complex effects on receptor density and responsiveness (see [98] for a review). It has also been shown that maternal smoking during pregnancy can affect offspring smoking behavior and cognitive performance in adolescence [99], [100]. Although there were no smoking habit differences between genotype groups in the present study, we were not able to control for potential effects of prenatal exposure to nicotine.

Although cognitive psychology has traditionally focused on the study of general mechanisms with within-subjects experimental designs, it has long been argued that individual differences can significantly contribute to revealing the structure of cognitive functions [101], [102]. The sequencing of the human genome has made available to the cognitive neuroscience community a vast pool of SNPs and other structural DNA variation [103]. The availability of this catalogue of naturally occurring individual variation encourages the convergence of correlational and experimental designs and makes it possible to study the molecular biology of attention and cognition in general [104], [105]. Results in the many studies published during the last decade show that the candidate gene association approach can be used to study the molecular biology of cognition. This is a non-invasive method that involves grouping participants based on genotype on a candidate single nucleotide polymorphism marker. If the current results can be generalized to other neurochemical systems and phenotypes, it should be relatively straightforward and efficient to test hypotheses about the neurochemical innervation of cognitive processes, provided that DNA and phenotypes from a sufficiently large and well-characterized sample has been obtained. Within the variety of attentional functions, SNP information from cholinergic and catecholaminergic system genes can be used to reveal (double) dissociations or interactive effects on specific subprocesses. The present results on the VS task and findings from other groups [45], [46], [82], suggests that modeling effects of two or more SNPs on separate subprocesses at multiple levels of load may prove to be fruitful.
